# IoU Regression with H+L-Sampling for Accurate Detection Confidence

**DOI:** 10.3390/s21134433

**Published:** 2021-06-28

**Authors:** Dong Wang, Huaming Wu

**Affiliations:** Center for Applied Mathematics, Tianjin University, Tianjin 300072, China; dong_wang@tju.edu.cn

**Keywords:** object detection, R-CNN, IoU regression, detection confidence, Non-Maximum Suppression

## Abstract

It is a common paradigm in object detection frameworks that the samples in training and testing have consistent distributions for the two main tasks: Classification and bounding box regression. This paradigm is popular in sampling strategy for training an object detector due to its intuition and practicability. For the task of localization quality estimation, there exist two ways of sampling: The same sampling with the main tasks and the uniform sampling by manually augmenting the ground-truth. The first method of sampling is simple but inconsistent for the task of quality estimation. The second method of uniform sampling contains all IoU level distributions but is more complex and difficult for training. In this paper, we propose an H+L-Sampling strategy, selecting the high and low IoU samples simultaneously, to effectively and simply train the branch of quality estimation. This strategy inherits the effectiveness of consistent sampling and reduces the training difficulty of uniform sampling. Finally, we introduce accurate detection confidence, which combines the classification probability and the localization accuracy, as the ranking keyword of NMS. Extensive experiments show the effectiveness of our method in solving the misalignment between classification confidence and localization accuracy and improving the detection performance.

## 1. Introduction

Object detection is one of the most fundamental and challenging problems in computer vision. It serves as a prerequisite for a wide range of downstream applications, such as instance segmentation [1], pose estimation [2], surveillance [3], and autonomous driving [4]. With the development of deep learning, remarkable progress in object detection has been made in recent years. Single-stage frameworks, such as RetinaNet [5] and Fcos [6], and two-stage frameworks, such as Faster R-CNN [7] and Cascade R-CNN [8], have substantially pushed forward the state of the art. Despite the apparent differences in the frameworks, object detection is usually formulated as two main tasks: One is a classification task to distinguish foreground from background and determine which category the object belongs to; the other is a localization task to regress a set of coefficients that localize the object as accurately as possible. For the duplicated detections matched with the same object, only the one with the highest score is considered true positive, others are considered false positive. However, in traditional Non-Maximum Suppression (NMS) to remove duplicated detections, the classification score is used as the ranking keyword, leading to a misalignment between the classification score and the localization accuracy. In this case, more accurately localized bounding boxes could be suppressed by less accurate ones but with higher classification scores.

Recently, a localization quality estimation branch, which is usually paralleled with the main branches of classification and bounding box regression, is introduced and leads to an encouraging advancement in the field of object detection. Fcos [6] and IoU-Net [9] predict the centerness score and the Intersection-over-Union (IoU) value, respectively, to estimate the localization quality. There are some differences between the two estimation scores. For the centerness score, it is used in anchor-free detectors to filter out the low-quality bounding boxes predicted by a location far from the center of an object. For the IoU value, it is used in anchor-based detectors to solve the misalignment between classification confidence and localization accuracy. At inference, the IoU is predicted on the detected bounding boxes, while the centerness is performed on each location irrelevant to the detected boxes. Thus, the IoU value between the detected bounding box and the match ground-truth is more correlated with the localization accuracy. In this paper, we focus on the estimation of IoU value and mainly study the sampling strategy for an estimation branch of IoU regression.

Currently, there exist two sampling strategies to train the IoU regression branch. First, ref. [10] uses the same training samples with the main branches of classification and bounding box regression. All three branches take samples from pre-defined anchor boxes. Second, ref. [9] adopts a uniform sampling w.r.t the IoU by manually augmenting the ground-truth to generate samples. The IoU branch and the two main branches are trained independently. For the existing strategies, we observe the following problems.

In the first case, the distributions of training and inference are consistent for the main branches but inconsistent for the IoU branch. In training, for each location on the feature map, the pre-defined anchor boxes are selected as training samples for the three branches. At inference, the three branches play different parts in the object detection pipeline. Specifically, given a set of anchors, the bounding box regression branch transforms the anchors to best fit the ground-truth; the classification branch distinguishes the categories of anchors through the probabilities for each class label; the IoU estimation branch predicts the quality of the detected bounding boxes. For classification and bounding box regression, there is consistency between training and inference distributions, which are all performed on the anchors. The consistency of distributions is a common paradigm in deep learning, which is a simple yet effective training strategy empirically. Following this paradigm, the consistency of distributions enables the network to efficiently learn good representations of a specific distribution consistent with inference. However, there is a discrepancy between training and inference distributions for the IoU regression branch which is performed on the detected bounding boxes at inference but trained on the anchors. This inconsistency inevitably induces ineffective learning.

In the second case, Ref. [9] first generates candidate samples by manually augmenting the ground-truth, and then it uniformly samples training examples from this candidate set w.r.t all IoU levels (>0.5). The manual augmentation assembles enough training examples for the uniform sampling. Compared with the uniform sampling from manual augmentation, the main tasks of classification and bounding box regression adopt random sampling from the Region Proposal Network (RPN) proposals. There is a dilemma that the training samples are generated by manually augmenting the ground-truth rather than using the RPN proposals. This brings three drawbacks: (1) The breaking of the unified training samples. The RPN proposals are used for both classification and bounding box regression, while extra samples are manually generated for training the IoU regression. (2) The ineffective learning of outlier. When all levels of samples are selected for the IoU regression, the samples of outliers, which are not consistent with the inference distribution, will lead to ineffective learning. (3) The increasing of learning difficulty. Ideally, the IoU regressor, based on the uniform sampling, is expected to be optimal at all IoU levels. However, the ideal regressor inevitably enhances the difficulty of learning.

One question is whether uniform sampling is necessary to train an IoU regressor. We analyze this question from two perspectives: (1) The intent of the IoU regression task. Different from the intent of bounding box regression which aims to be infinitely close to the target box, the objective of the IoU value is to distinguish which has more accurate localization for the two overlapping bounding boxes in the NMS procedure. In other words, rather than the accurate IoU value, the IoU regressor is intent to have the characteristic of distinction between two overlapping boxes. (2) The effectiveness of a single IoU regressor to all IoU levels. In Cascade R-CNN [8], it suggests that each bounding box regressor performs best for the corresponding IoU level that the regressor was trained. A cascaded regression is proposed, such that the regressors deeper into the cascade are sequentially optimized to higher IoU levels. Thus the difficulty of bounding box regression to different IoU levels is decomposed into a sequence of stages. For a single regressor, the light head is usually framed as two fully connected layers, resulting into that the capacity of learning is limit. Compared with the cascaded bounding box regressors, the learning of good representations for all IoU levels is more difficult for a single and light IoU regressor. In conclusion, the easier task of IoU regression uses a more complex strategy of uniform sampling. For the single IoU regressor, it is uncertain whether the uniform sampling leads to effective learning for all IoU levels.

In this work, we aim to improve the performance of the two-stage Faster R-CNN by an additional IoU regression task and solve the mentioned sampling problems for the IoU regression branch. To distinguish from the two main tasks, the IoU regression task is also called the auxiliary task. Based on the fact that the IoU regressor is operated on the detected bounding boxes (the RPN proposals after regressed) at inference, sampling from the regressed RPN proposals can guarantee the consistent distributions for training and inference. Thus, we can solve the inconsistency in the first sampling case. It is worth noting that the regressed RPN proposals are heavily titled toward high IoU levels and the RPN proposals toward low IoU levels. When sampling from the regressed RPN proposals, the IoU regressor can learn good representations for high IoU distribution consistent with inference. Ideally, the IoU regressor should be optimal at all IoU levels. However, the uniform sampling by manually augmenting the ground-truth, which inevitably enhances the difficulty of learning, is uncertainly effective to all IoU levels. A compromise of uniform sampling is selecting low IoU samples and high IoU samples to train the branch of IoU regressor, resulting in an IoU regressor that is optimal at not only high IoU levels but also low IoU levels in a single structure. In this manner, we can reduce the learning difficulty in the second case.

In this paper, we introduce an auxiliary IoU regression branch based on Faster R-CNN, which is called IoU-Aware R-CNN. We propose an H+L-Sampling strategy to select the high and low IoU samples simultaneously, in which the low IoU samples are selected from the RPN proposals and the high IoU samples are obtained by transforming the low IoU samples. The high IoU samples satisfy the consistent sampling. On the basis of the consistent samples, adding the existing low IoU samples brings negligible computation burden and can still substantially improve the performance and robustness. This strategy inherits the effectiveness of consistent sampling and reduces the difficulty of uniform sampling, resulting in an IoU regressor that is optimal at both low and high IoU levels. This simple but powerful branch demonstrates significant improvement in detection performance. Our IoU regression is still powerful when trained on few samples, which requires few computational resources and more compatible with real-world applications. The probabilities of categories reflect classification confidence, and the predicted IoU values between the detected bounding boxes and the ground-truth reflect localization confidence. Finally, We combine the predicted IoU with the probability as the final detection confidence for the rank process of NMS, removing duplicated bounding boxes and preserving accurately localized bounding boxes.

In summary, the main contributions of this paper are listed as follows:

- We propose an H+L-Sampling strategy, which satisfies the consistency of distributions and the low difficulty of learning, to train an additional IoU regression branch in our IoU-Aware R-CNN. For the auxiliary task, our sampling is simple and effective to estimate the localization accuracy.
- In the whole of our IoU-Aware detector, we have a unified structure in both training and inference. Rather than manually augmenting the ground-truth, all three branches take samples from the RPN proposals in training. In the post-process of NMS, the detection confidence is proposed, which encodes the probability of that class appearing in the box and the accuracy of the predicted box localizing the object, simultaneously.
- Extensive experiments show the effectiveness of our sampling strategy to solve the problem of absent localization accuracy, as well as its simplicity but competitiveness even compared with several state-of-the-art object detectors. Due to its effectiveness and simplicity, our IoU regression branch can be compatible with most two-stage detectors.

## 2. Related Work

### 2.1. The Consistent Sampling on Reference Boxes or Points in Object Detection

Object detection deals with detecting multi-scale objects of a certain class in an image. In modern deep learning based detectors, the key idea is to pre-define a set of reference boxes at different locations of the image. The novel anchor box was firstly introduced in the two-stage Faster R-CNN [7] serving as reference boxes at multiple scales and aspect ratios on the single-scale feature map. The concept of anchor box is adopted in many subsequent detectors, such as FPN [11], SSD [12], DSSD [13], YOLO9000 [14], and RetinaNet [5], in which the anchor boxes are defined on the feature pyramid. Instead of using anchor boxes, YOLO [15] predicts bounding boxes at the grid cells near the center of objects. In YOLO, the grid cells are viewed as reference boxes with respect to objects. Recently, many anchor-free detectors [6,16,17,18] are proposed to eliminate the pre-defined set of anchor boxes. In these methods, rather than using the anchor boxes or the grid cells, the detectors directly predict bounding boxes at each location of the convolutional feature in different ways. At each location, CornerNet [16] detects a pair of corners of a bounding box and groups them to form the final detected, and Fcos [6] directly predicts the bounding boxes. For the all mentioned detectors, there is a similarity that the references of boxes or points are pre-defined to locate the objects.

In the one-stage detectors, the main branches of classification and bounding box regression take training samples from the references of boxes or points, and the two main branches are performed on the consistent references during inference. The difference of two-stage detectors is that a second stage further refines the RPN proposals and satisfies consistent sampling during training and inference. For the two main branches, the most popular sampling strategy is random sampling with a fixed positive-to-negative ratio. Besides, more effective methods are proposed. The hard mining of OHEM [19], Libra R-CNN [20], and RetinaNet [5] select hard samples. PISA [21] indicates that prime samples, the positive samples with the highest IoUs and the negative samples with the highest scores, are important for training an object detector. Regardless of sampling strategies, there is a consistency between training and inference distributions, which enables the detector to effectively learn good representations of a specific distribution consistent with inference.

### 2.2. The Estimation of Localization Quality

In NMS with absent localization accuracy, the score of localization is determined by the classification score to rank all detections, suppressing the highly overlapping detections with a low score. The absence of localization accuracy leads to accurately localized boxes being suppressed by less accurate ones. A recent trend of object detectors is to introduce an additional branch to estimate the quality of localization, which brings significant improvements in detection accuracy. Fcos [6] predicts the centerness score to filter out the low-quality bounding boxes which are far from the center of an object. In [9,10,22], the IoU between the detected bounding box and the corresponding ground-truth is used to estimate the localization quality. In Mask Scoring R-CNN [23], a MaskIoU branch is added to predict the IoU between the predicted mask and the ground-truth mask. Thus the quality estimation can be combined with classification score as more accurate detection confidence for the rank process of NMS, preserving more accurately localized boxes.

In the one-stage Fcos [6], the quality estimation branch is trained and tested on the reference points, the locations of the convolutional feature. However, in [10], the estimation branch is trained on the anchors but is performed on the detected bounding boxes in inference, leading to a discrepancy between training and inference distributions. In the training of the MaskIoU branch [23], it takes training samples from RPN proposals, and the concatenation of the feature from RoIAlign layer and the final predicted mask is used as the input of MaskIoU head. Thus the MaskIoU head merges the information of the predicted mask together to regress its IoU with the ground-truth mask. In IoU-Net [9], the training examples are manually generated by augmenting the ground-truth, instead of taking from RPN proposals. Specifically, it utilizes a set of randomized parameters to transform all ground-truth bounding boxes, resulting in a candidate bounding box set. In order to be robust to the change of the input distributions with different detectors, [9] takes uniform samples w.r.t all IoU (>0.5), which makes it difficult to train the IoU branch that can be effective to all distributions. Different from the above methods, our H+L-Sampling has consistent distinctions between training and inference for the IoU branch. Compared with the manually uniform sampling, our IoU regressor, which is optimal at high and low IoU levels, is trained in a more simple way.

## 3. Proposed Method

**Figure 1:**
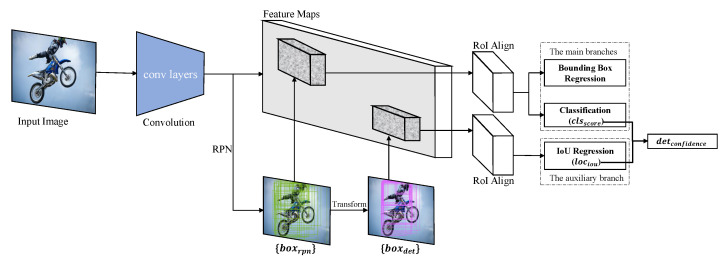
The framework of IoU-aware R-CNN. The main branches and the auxiliary branch are performed on $\left\{box_{rpn}\right\}$ and $\left\{box_{det}\right\}$, respectively. The auxiliary branch estimates the localization quality and almost does not affect the original network of Faster R-CNN, which only changes the ranking confidence of the NMS process. Unless otherwise stated, we use class-agnostic IoU regression in a simple manner.

In this section, we propose the IoU-Aware R-CNN, adding an auxiliary branch of IoU regression based on Faster R-CNN, as shown in Figure 1. The auxiliary branch of IoU regression is paralleled from the main branches of classification and bounding box regression. The three branches play different parts in the two-stage detection pipeline. Specifically, given a set of RPN proposals $\left\{box_{rpn}\right\}$, the bounding box regressor transforms the RPN proposals to best fit the ground-truth; the classifier distinguishes the categories of the RPN proposals through the softmax probabilities for each class label; different from the main branches which are performed on the RPN proposals, the IoU regressor predicts the quality of the detected bounding boxes $\left\{box_{det}\right\}$. Thus, for each detected bounding box, there are two confidences to reflect the performance of detection: The classification score ($cls_{score}$) indicates the probability of which category the bounding box belongs to; the localization IoU ($loc_{iou}$) with the corresponding ground-truth indicates the localization accuracy of the bounding box. The multiplication of the two confidences is used as the final detection confidence ($det_{confidence}$) for the rank process of NMS during inference. In the following, we show the details of our method.

### 3.1. Separate Sampling for the Main and Auxiliary Branches

**Figure 2:**
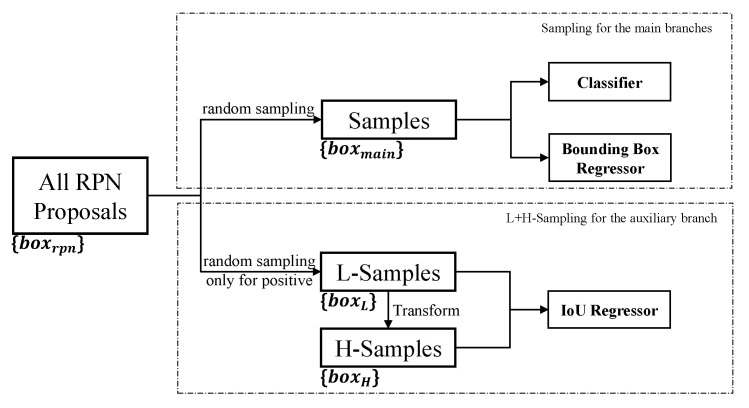
For the main branches and the auxiliary branch, we separately take samples from the RPN proposals $box_{rpn}$, adopting different sampling strategies. Note that the Transform refers to using the bounding box regressor in the main branches to refine the L-Samples and obtain the H-Samples.

In our IoU-Aware R-CNN, the sampling of the auxiliary branch of IoU regression is separate from the sampling of the two main branches, shown in Figure 2. There are two reasons for this design: (1) The most widely adopted sampling method for the two main branches is the random sampling with a fixed positive-to-negative ratio, like in Faster R-CNN [24]. Besides, more efficient sampling strategies are proposed to improve the performance. One popular idea is hard mining to select hard samples, such as OHEM [19], Libra R-CNN [20], and RetinaNet [5]. Recently PISA [21] focus on the prime samples which have a greater influence on the performance of object detection. Using separate sampling for IoU regression makes the auxiliary branch more compatible with these detectors that adopt the mentioned sampling strategies for the main branches. (2) When using the separate sampling, it is convenient for us to study the ablation experiments and design a simple sampling only focusing on the positive for IoU regression task.

For the main branches of classification and bounding box regression, we adopt the random sampling following Faster R-CNN. For all RPN proposals, they highly overlap with each other. Before sampling, the NMS with a fixed IoU threshold of 0.7 is adopted to reduce redundancy, getting the set of RPN proposals $\left\{box_{rpn}\right\}$ in Figure 2. Note that the IoU regression branch also selects samples from the same candidate set $\left\{box_{rpn}\right\}$. For each $box_{rpn}$, if the IoU with the ground-truth is greater than 0.5, we assign a positive label to $box_{rpn}$. Otherwise, we assign a negative label. Finally, we randomly select positive and negative samples $\left\{box_{main}\right\}$ with a fixed positive-to-negative ratio.

**Figure 3:**
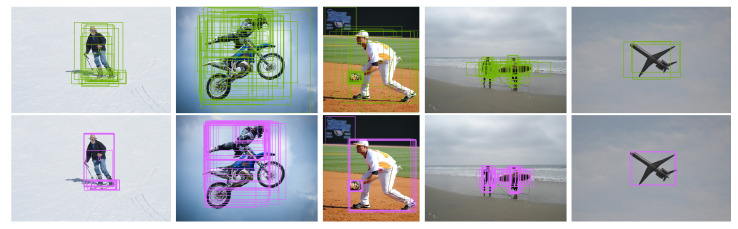
$box_{rpn}$ (**top**) vs. $box_{det}$ (**bottom**). The distributions of samples are intuitively different, and the regressed $box_{det}$ have higher IoU with objects than $box_{rpn}$.

For the auxiliary branch of IoU regressor, it is operated on the detected bounding box to predict its IoU value with the corresponding ground-truth at inference. Corresponding to Figure 1, given the set of RPN proposals $\left\{box_{rpn}\right\}$, the detected bounding boxes $\left\{box_{det}\right\}$ can be computed by:

(1) $$box_{det}=transform\left(box_{rpn},c\right),$$

where transform is the bounding box regressor taking *c* as parameters. The core idea of a bounding box regressor is that a network directly learns to transform a bounding box to its designated target. Inspired by the observation in Cascade R-CNN [8] that the output IoU of a bounding box regressor is almost invariably higher than the input IoU, the regressed $box_{det}$ would have a higher IoU level than $box_{rpn}$. We show some qualitative results of the head-to-head comparison in Figure 3.

For the IoU regression task, we propose a simple and effective strategy in Figure 2, H+L-Sampling, to select high and low IoU samples simultaneously and train a single IoU regressor. First, we adopt the mentioned random sampling to only select positive samples from $\left\{box_{rpn}\right\}$ and obtain the samples with low IoU $\left\{box_{L}\right\}$. Then Equation (1) is performed on $\left\{box_{L}\right\}$ to get the high IoU samples $\left\{box_{H}\right\}$ which is a small part of $\left\{box_{det}\right\}$, satisfying the distribution consistency between training and inference for IoU regression. Finally, the two sets of samples are used to train a single IoU regressor. Note that multi-stage IoU regressors like cascaded structure in [9] are unavailable for the task of IoU evaluation. Because, even if cascaded IoU regressors are separately trained by $\left\{box_{L}\right\}$ and $\left\{box_{H}\right\}$, the IoU evaluation is performed on the final detections $\left\{box_{det}\right\}$ and the same cascade procedure is inapplicable at inference.

We analyze the effectiveness of our proposed H+L-Sampling strategy: (1) $\left\{box_{L}\right\}$ is the most convenient and straight samples focusing on the low IoU distribution. While there is a small discrepancy between training and inference distributions, $\left\{box_{L}\right\}$ are feasible to train the IoU regressor in a simple manner. (2) $\left\{box_{H}\right\}$ focuses on high IoU distribution which is consistent with inference. This sampling guarantees the effective learning of a specific distribution consistent with inference. It is worth noting that the consistency means that the input distributions of both training and inference tend toward a specific IoU level but there might exist lower or higher IoU examples. Under the same number of samples, the consistent samples of $\left\{box_{H}\right\}$ is a better choice. (3) $\left\{box_{L}\right\}+\left\{box_{H}\right\}$ can be regarded as adding low IoU distribution on the basis of the consistent distribution, resulting in the diversity of samples. Ideally, the complete samples of training should include all IoU levels that maybe appear at inference. However, training samples are more, more difficult for learning. It is a trade-off between the diversity of samples and the difficulty of learning. Our H+L-Sampling, which selects the more effective samples $\left\{box_{H}\right\}$ and takes full advantage of the existing samples $\left\{box_{L}\right\}$, is simple and effective.

Compared with the manually uniform sampling in [9,22], our proposed sampling differs from it in that: (1) In uniform sampling, manually augmenting the ground-truth is used to generate enough samples for each IoU interval. In our method, we take samples from the RPN proposals as same as the two main branches, resulting in a more unified sampling for the detector. (2) In the manual samples, the number of outliers that are not consistent with the inference distributions is more than the RPN proposals. While the RPN proposals are not completely consistent, the distribution difference between training and inference is smaller compared with the manual samples. (3) For the uniform sampling in [22], it divides the IoU into 4 intervals, and each ground-truth keeps 64 samples for each IoU interval. Given an image with *K* annotated ground-truths, the overall number of training samples is K*4*64. However, in default settings of our sampling, the number of RPN proposals and regressed RPN proposals is 2*64, which are much fewer samples to train the IoU branch. The corresponding difficulty of learning is much reduced. (4) For our H+L-Sampling, it can be seen as two intervals: Low IoU level and high IoU level. For each interval, the only difference between the uniform sampling and the proposed sampling is the number of samples (K*64 vs. 64). In our sampling, it is interesting to observe that when changing the sample number (64) to 32 or 128, it just has a difference of 0.1, which suggests that the IoU evaluation task, aimed to distinguish the localization accuracy of two overlapping bounding boxes, is insensitive to the number of samples. The insensitivity reflects that the dense samples (K*64) for each interval in uniform sampling are unnecessary for IoU regression.

### 3.2. Loss Function

For the two main branches, the classifier h(x) assigns the candidate bounding box *x* to one of the categories including background and the regressor f(x) regresses the parameterized coordinates of the target bounding box associated with the candidate. Given the training set $\left\{x,p^{*},t^{*}\right\}$, the loss function for the main branches follows Fast R-CNN [24]:

(2) $$\begin{array}{c} L_{main}\left(x,p^{*},t^{*}\right)=\sum_{i}\left(L_{cls}\left(h\left(x_{i}\right),p_{i}^{*}\right)+L_{reg}\left(f\left(x_{i}\right),t_{i}^{*}\right)\right). \end{array}$$

Here, *i* is the index of a training sample in a mini-batch, $p_{i}^{*}$ is the class label of *x*, and $t_{i}^{*}$ is the 4 parameterized coordinates of the ground-truth. The classification loss $L_{cls}$ is the cross-entropy loss $CE\left(p,p^{*}\right)=-\sum_{k}p_{k}^{*}log\left(p_{k}\right)$, where *k* is the index of categories. The regression loss $L_{reg}$ uses the $L_{1}$ loss following the default setting in mmdetection [25].

For the auxiliary branch, the regressor g(x) regresses the target IoU value between the candidate bounding box *x* and the corresponding ground-truth. Given the low IoU training set $\left\{x_{L},u_{L}^{*}\right\}$ and the high IoU set $\left\{x_{H},u_{H}^{*}\right\}$, the loss function is defined as:

(3) $$\begin{array}{c} L_{aux}\left(x_{L},x_{H},u_{L}^{*},u_{H}^{*}\right)=\sum_{j}\left(L_{reg}\left(g\left(x_{L,j}\right),u_{L,j}^{*}\right)+\lambda L_{reg}\left(g\left(x_{H,j}\right),u_{H,j}^{*}\right)\right). \end{array}$$

Here, *j* is the index of a training sample in a mini-batch, $u_{L,j}^{*}$ and $u_{H,j}^{*}$ are the target IoU of $x_{L,j}$ and $x_{H,j}$, respectively. Note that two training sets are used to optimize a single regressor. The losses of low and high IoU samples are weighted by a balancing parameter λ. By default we set λ=2, focusing more on the consistent samples which are more effective.

Overall, we use a multi-task loss to jointly train the main and auxiliary branches:

(4) $$\begin{array}{c} L=L_{main}+L_{aux}. \end{array}$$

For bounding box regression, the numerical value of $t_{i}^{*}$ can be very small, and $t_{i}^{*}$ is usually normalized by its mean and variance to improve the effectiveness of learning. For IoU regression, we note that the normalization is not required and could be simplified. The loss of $L_{main}$ to $L_{aux}$ ratio is roughly 4:1. Due to the small weight of $L_{aux}$, the auxiliary branch almost does not affect the original outputs of the detector, which is more compatible with practical applications.

### 3.3. Detection Confidence for NMS

**Figure 4:**
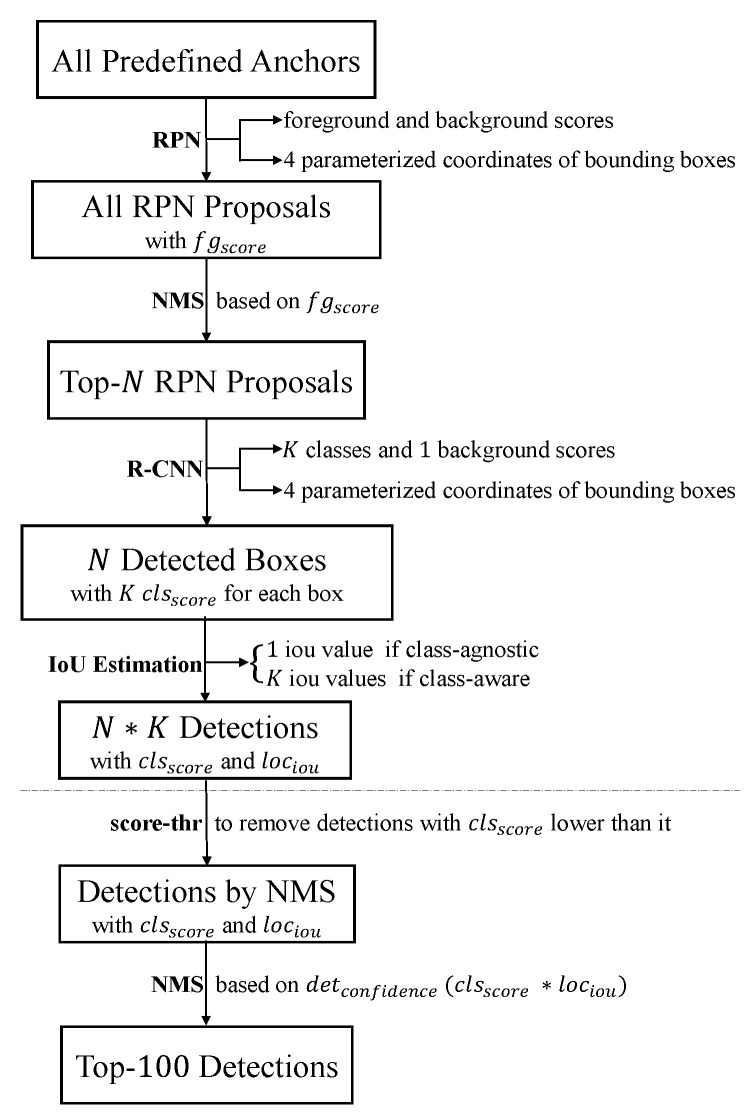
The overall pipeline of our two-stage detector of IoU-Aware R-CNN. We change the way to choose the top-N detections. This simple but powerful branch demonstrates significant improvement in detection performance.

At inference, the overall pipeline of our two-stage detector of IoU-Aware R-CNN is shown in Figure 4. In the first stage of RPN, each anchor generates an RPN proposal with a foreground score ($fg_{score}$), and NMS based on the $fg_{score}$ is performed on all RPN proposals to choose top-N RPN proposals for detection. In the second stage of R-CNN, the top-N RPN proposals are refined by the four parameterized coordinates and generate N detected boxes with K classification scores ($cls_{score}$) for each box. Then the IoU estimation is performed on the N detected boxes to predict their localization IoU ($loc_{iou}$) with the corresponding ground-truth. If the IoU regressor is class-agnostic, the K classes correspond to the same $loc_{iou}$; if class-aware, each of the K classes corresponds to its own $loc_{iou}$. For the N detected boxes with K classes, the detector finally outputs N×K(1000 × 80 = 80,000) detections with the classification confidence $cls_{score}$ and the localization confidence $loc_{iou}$. Note that if the bounding box regressor is class-aware, each of the K classes gets its own coordinates and the corresponding parameterized coordinates for each class are used to refine the RPN proposal, generating N×K detected boxes of which the difference between classes is slight. If we perform the IoU estimation on the N×K detected boxes, it will bring significant computation cost. So the IoU estimation is on the *N* detected boxes with the maximum classes score, which almost has no influence on the detection performance.

The post-processor of NMS aims to remove duplicated detections and choose the top-100 final detections which are used to evaluate the detection performance. Before NMS, a threshold of score_thr is usually used to remove detections with scores lower than it. Note that there still exist a lot of detections with relatively low $cls_{score}$, which cannot be distinguished only by the $loc_{iou}$. So we define detection confidence as:

(5) $$\begin{array}{c} det_{confidence}=cls_{score}*loc_{iou}, \end{array}$$

which encodes both the probability of that class appearing in the predicted bounding box and how well the bounding box fits the object. The detection confidence $det_{confidence}$ is used as the metric for ranking detections, and the suppression of duplicated detections is aware of the localization accuracy and the classification probability. Finally, NMS based on the ranking keyword of $det_{confidence}$ is used to choose the top-100 detections, preserving detections with a more accurate localization. The difference of soft-NMS is replacing box elimination by the decrement of confidence. So the $det_{confidence}$ is also suitable for soft-NMS and we show consistent improvements by experiments.

## 4. Experiments

### 4.1. Datasets

We adopt MS COCO 2017 [26] as the primary benchmark for all experiments since it is the most widely used dataset for object detection. In MS COCO, it contains objects at a wide range of scales, including a high percentage of small objects. Objects are less iconic and amid clutter or heavy occlusion, which is more challenging. We perform training on the 118 k images of the COCO 2017 train set. In ablation, evaluation is done on the 5 k images of the COCO 2017 validation set. We report the results on the 20 k images of the COCO 2017 test-dev set (without public labels). To demonstrate the generalization ability, we also show the results on PASCAL VOC [27]. In PASCAL VOC, the union of VOC 2007 trainval and VOC 2012 trainval are used for training, and the results are evaluated on VOC 2007 test. For all experiments, we report average precision (AP) over multiple IoU thresholds (from 0.5 to 0.95) to measure the detection performance.

### 4.2. Implementation Details

All experiments are implemented on MMdetection [25], an object detection toolbox that provides a flexible toolkit to reimplement existing methods and develop our own detectors. If not otherwise specified, we use the following settings. The input images are resized to a maximum scale of 1333 × 800 without changing the aspect ratio. We use 4 TITAN X GPUs for training with a total batch size of 8 (2 images for GPU). There is no data augmentation to optimize the overall objective. In our experiments, two training schedules are adopted: “1x” and “2x”, which means 12 epochs and 24 epochs, respectively, in training. In “1x”, the learning rate is dropped 10 times at the 8th and 11th epochs. In “2x”, the learning rate is dropped at 16th and 22th epochs. The learning rate is initialized to 0.01, the weight decay and momentum to 0.0001 and 0.9, respectively. Unless otherwise stated, we adopt the “1x” training schedule for most of the following studies. We set score_thr to 0.05 following the default hyper-parameter in MMDetection. All samples for the three branches are from the RPN proposals without manual augmentation from the ground-truth. The branches of classification and localization sample the RPN proposals following Faster R-CNN, in which 512 proposals are selected including foreground and background. For the IoU branch, $\left\{box_{L}\right\}$ are selected from positive RPN proposals, and $\left\{box_{H}\right\}$ are obtained by transforming $\left\{box_{H}\right\}$. For the two sets, we set the default number of samples to 64.

### 4.3. Ablation Study

All the ablation study experiments are based on ResNet-50 [28] backbone. Class-agnostic IoU regressor is used in a simple manner.

(1) The Choices of Training Samples: Table 1 summarizes the performance of different samples for IoU regression. The simplest sampling of $\left\{box_{L}\right\}$ directly takes samples from the RPN proposals and brings improvements from 37.7 (baseline FPN) to 38.5. It suggests that the samples RPN proposals are also feasible to train the IoU predictor in a simple manner. However, there is a small problem that the IoU predictor is performed on the detected bounding boxes rather than RPN proposals in inference. This discrepancy between training and inference distributions inevitably induces ineffective learning. We address this problem by selecting $\left\{box_{H}\right\}$ which is obtained by transforming $\left\{box_{L}\right\}$ and has consistent distribution with the detected bounding boxes. In this manner, a further gain of 0.3 point is attained, proving that consistent sampling is a simple yet effective strategy for the training process. The distributions of $\left\{box_{L}\right\}$ and $\left\{box_{H}\right\}$ are heavily titled toward low IoU level and high IoU level, respectively. The corresponding IoU regressors are optimal at the corresponding IoU levels. When the low and high samples are simultaneously used to train the IoU regressor, the H+L-Sampling strategy further improves the performance by 0.2 point and achieves the best result of 39.0 AP.

(2) Number of Samples: The impact of the number of samples for IoU regression is summarized in Table 2. We can reach 39.0 Ap in the default setting 64. As we increase the number to 128 or decrease to 32, it just has a difference of 0.1 which is not significant, shown that our IoU regression branch is insensitive to the number of samples. Our IoU regression is still powerful when trained on few samples (32), which requires few computational resources.

We analyze this insensitivity from two aspects: (a) The H+L-Sampling Strategy. In this manner, we select the high and low IoU level samples to train the IoU regressor. The operation of selecting the high IoU samples is aimed to satisfy the condition of consistent distributions between training and inference. Meanwhile, this operation could increase the number of training samples. (b) The Intent of Task. For the task of IoU regression, the predicted IoU, an interpretable indicator of the localization quality on each bounding box, is used to distinguish which has more accurate localization for two overlapping bounding boxes. It is unnecessary to be infinitely close to target as the task of bounding box regression. In conclusion, the H+L-Samples can implicitly increase samples and the task of IoU regression is intent to have the characteristic of distinction between two overlapping detected boxes, resulting in the insensitivity of IoU regression.

### 4.4. Main Results

We report our results on different backbones to prove the effectiveness of our method. All settings are as above.

(1) Detection Performance and Inference Speed: The IoU-aware R-CNN based on three popular backbones are compared in Table 3. For the baseline FPN, it uses a classification score ($cls_{score}$) to rank all detected bounding boxes in NMS. Compared with FPN, when our IoU-aware R-CNN also uses $cls_{score}$ as the ranking keyword, there is just a difference of 0.1~0.2. This slight difference indicates that our auxiliary IoU regression branch has little influence on the main branches, which makes the auxiliary branch more likely to be compatible with other detectors. When using the introduced detection confidence ($det_{confidence}$), the IoU-Aware R-CNN improves on these FPN baselines consistently by 1.3~1.6 points. These results suggest that our IoU-Aware R-CNN is widely applicable across light or deep backbones.

At inference, the IoU branch predicts the IoU between the detected bounding box and the ground-truth, then using the combination of $cls_{score}$ and $loc_{iou}$ defines $det_{confidence}$ to rank all detections in the NMS procedure. For the auxiliary branch based on the three backbones, the computational cost is small, as shown in Table 4.

(2) The effect on soft-NMS: The only difference between NMS and soft-NMS is replacing the box elimination with the decay of score. Our introduced confidence of $det_{confidence}$ is also suitable for soft-NMS and Table 5 shows consistent improvements on the baseline FPN by 1.3~1.5 points. Compared with NMS, when $det_{confidence}$ is used in soft-NMS, the gains slightly decrease on the whole of three backbones. To explain the phenomenon, we detailedly review which boxes are preserved after NMS and soft-NMS in the following.

NMS starts with detection boxes *B* with scores *S*. After selecting the detection *M* which has the maximum score, it removes any neighboring box *b* with an overlap greater than a fixed threshold with *M*. In the soft-NMS procedure, decaying the scores leads to preserving two kinds of more boxes: the first kind is that *b* contains an object not covered by *M*, which would decrease the miss-rate of detection; the second kind is that *b* is duplicated box and cover the same object with *M*. For the duplicate box *b*, if the ranking is correct and *M* has more accurate localization than *b*, it would generate a false positive. Actually, the classification score cannot correctly reflect the accuracy of localization, and if *b* is more accurate than *M*, it will not lead to a miss at a higher detection threshold. Specifically, We hypothesis that *M* reaches the level metrics of AP${}_{k}$, which means that the IoU between *M* and the ground-truth is higher than *k* and lower than the next level. If the duplicate box *b* has lower localization accuracy than *M*, it will be considered as false positive for the metric of AP${}_{k}$ and the lower metrics. If *b* has higher accuracy of localization, it may be true positive for the metrics higher than AP${}_{k}$. Soft-NMS could preserve the detected bounding boxes with a low classification score but high accurate localization, which is helpful to measure AP over multiple overlapping thresholds. By preserving more detections, the soft-NMS solves the problem of absent localization accuracy to some extent. When using $det_{confidence}$ in both NMS and soft-NMS, this confidence is helpful for selecting the high accurate bounding boxes, resulting in that the improvement of soft-NMS slightly decreases.

(3) Results on PASCAL VOC: We further experiment on the PASCAL VOC dataset following the default setting of mmdetection. The training differences with the COCO dataset are that the learning rate is initialized to 0.005 and dropped at 9th epoch. The detection results in Table 6 show that the IoU-Aware R-CNN also has significant improvements over multiple backbones on PASCAL VOC. On this relatively easy dataset, it is more necessary to choosing the top-N detections using the ranking confidence aware of localization.

### 4.5. Comparison with Other Methods

For better performance, we adopt “2x” training schedule and class-aware IoU regressor for the following experiments.

(1) Comparison with IoU-Net [9]: In [9], the IoU regressor is independent of specific detectors. When cooperates with different detectors, the IoU predictor should be robust to the change of the input distributions. For this purpose, [9] first uses manually augmenting the ground-truth to generate candidate samples and then adopts uniform sampling w.r.t the IoU to select training data from this candidate set. Based on this manner of training, it is expected that the IoU predictor is effective to all IoU levels, which inevitably enhances the difficulty to train the IoU regression branch. Different from [9], the IoU predictor of our method is dependent on specific detectors without the change of the input distribution. Our H+L-Samping only selects the high IoU samples and the low IoU samples, which reduces the degree of difficulty to train the IoU regression branch. Table 7 indicates that our method can effectively solve the misalignment of classification confidence and localization accuracy. Compared with the 2.1% improvement on IoU-Net (with a bounding box refinement), our method, based on a stronger FPN, can achieve a 2.2% improvement. When setting the score_thr from 0.05 to 0.001, more detection boxes are in the candidate list of NMS. Our method further improves the performance by 0.4 point, reaching the best result of 42.0 AP.

(2) Comparisons on COCO test-dev: In Table 8, we present comparisons of our detection method, adding an auxiliary branch of IoU regression on FPN, with existing detectors on MS COCO test-dev. The first group of detectors in Table 8 are the one-stage, the second group is the two-stage, and the last group is our method on different backbones. It is noted that all detectors of our method use the single-scale training strategy, while most recently one-stage methods adopt the multi-scale training for better performance. Our method with ResNet-101 achieves 42.3 Ap, which is superior to most of the existing detectors with the same backbone, including the one-stage methods of FSAF (40.9), FCOS (41.5), FoveaBox (40.8), and the two-stage of Libra R-CNN (41.1),Grid R-CNN (41.5). Compared with Cascade R-CNN and TridentNet (MS${}_{train}$) which have more complex head structures, there is just a difference less than 0.5. Based on the backbone of ResNeXt [29], our method obtains 43.4 AP, which has better performance than PISA (42.3). Further, when soft-NMS is employed and trainval dataset is used to train the detector, IoU-Aware R-CNN${}^{*}$ can obtain our best performance of 44.3 AP in the case of single-scale training and inference. Compared with LTM and ATSS adopting multi-scale training, the difference with our best performance on single-scale training is less than 0.8 point. Consider the simplicity of our auxiliary branch, it also shows the effectiveness of our method.

## 5. Conclusions

In this paper, we propose an H+L-Sampling strategy, including high and low IoU samples, to effectively train the IoU regression branch for accurate ranking confidence of NMS. Based on the fact that the IoU regressor is operated on the detected bounding boxes rather than the RPN proposals, the high IoU samples enable a consistent distribution between training and inference. For the low IoU samples, although there is a small discrepancy between training and inference distributions, it is feasible to train the IoU regressor in a simple manner. The H+L-Sampling can be regarded as adding low IoU samples on the basis of the consistent high IoU samples, which brings negligible computation burden and results in a more effective way of training. Compared with the uniform sampling w.r.t all IoU levels, our method reduces the degree of training difficulty and completes the objective that the predicted IoU is enabled to distinguish the localization accuracy of two overlapping bounding boxes. Finally, we introduce the detection confidence encoding the classification probability and localization accuracy simultaneously. Extensive experiments on different architectures have shown that our method can substantially improve detection performance, especially under high IoU metrics.

## Figures and Tables

**Table 1:** Results of selecting different training samples for IoU regression. Quantitative results show that our H+L-Sampling strategy is effective to resolve the misalignment problem.

Method	AP	AP${}_{50}$	AP${}_{60}$	AP${}_{70}$	AP${}_{80}$	AP${}_{90}$
Baseline FPN	37.7	58.5	54.2	46.4	33.2	11.1
$\left\{box_{L}\right\}$	38.5	57.7	53.4	46.9	35.4	14.5
$\left\{box_{H}\right\}$	38.8	57.9	53.7	47.1	35.5	15.2
$\left\{box_{L}\right\}+\left\{box_{H}\right\}$	39.0	57.8	53.6	47.2	36.1	15.5

**Table 2:** The impact of the number of samples for IoU regression.

Numbers	AP	AP${}_{50}$	AP${}_{60}$	AP${}_{70}$	AP${}_{80}$	AP${}_{90}$
32	38.9	57.8	53.5	47.1	36.1	15.3
64	39.0	57.8	53.6	47.2	36.1	15.5
128	39.1	57.9	53.8	47.2	36.4	15.5

**Table 3:** Detailed comparison of NMS with different confidences of $score_{cls}$ and $det_{confidence}$ on multiple popular backbones.

Backbone	Method	NMS$\_{\mathrm{cls}}_{\mathrm{score}}$	NMS$\_\det_{\mathrm{confidence}}$	AP	AP_50_	AP_60_	AP_70_	AP_80_	AP_90_
R-50	FPN	✓		37.7	58.5	54.2	46.4	33.2	11.1
	IoU-aware	✓		37.6	58.2	53.6	46.2	33.4	11.7
	R-CNN		✓	39.0	57.8	53.6	47.2	36.1	15.5
R-101	FPN	✓		39.4	60.1	55.6	48.3	35.6	12.9
	IoU-aware	✓		39.6	60.0	55.6	48.3	36.1	13.6
	R-CNN		✓	41.0	59.7	55.5	49.2	38.8	17.7
X-101-32x4d	FPN	✓		41.2	62.2	57.8	50.6	37.8	14.6
	IoU-aware	✓		41.3	62.0	57.6	50.4	38.3	14.9
	R-CNN		✓	42.6	61.6	57.3	51.2	40.4	19.1

**Table 4:** Inference speed of different backbones on a single TITAN X GPU.

Backbone	R-50		R-101		X-101-32x4d	
IoU regression	✗	✓	✗	✓	✗	✓
Speed (sec./image)	0.114	0.134	0.149	0.175	0.185	0.213

**Table 5:** The effect of detection confidence on soft-NMS.

Backbone	R-50		R-101		X-101-32x4d	
IoU regression	✗	✓	✗	✓	✗	✓
NMS	37.7	39.0 (↑1.3)	39.4	41.0 (↑1.6)	41.2	42.6 (↑1.4)
soft-NMS	38.3	39.6 (↑1.3)	40.1	41.6 (↑1.5)	42.0	43.3 (↑1.3)

**Table 6:** Detection results on PASCAL VOC 2007 test.

Backbone	IoU Regression	Speed (sec./Image)	AP	AP_50_	AP_60_	AP_70_	AP_80_	AP_90_
R-50	✗	0.079	50.4	80.1	74.5	64.3	43.0	11.2
	✓	0.102	54.1	80.5	76.0	66.1	48.9	18.6
R-101	✗	0.102	54.3	82.1	77.8	67.9	48.1	16.1
	✓	0.125	56.8	81.3	77.4	68.8	53.3	23.2

**Table 7:** Comparison with IoU-Net [9] on MS COCO validation. Ours${}^{+}$ means that the result is evaluated on a smaller score_thr of 0.001, resulting in more detection boxes in the candidate list of NMS.

Method	AP	AP${}_{50}$	AP${}_{60}$	AP${}_{70}$	AP${}_{80}$	AP${}_{90}$
FPN [9]	38.5	60.3	55.5	47.6	33.8	11.3
IoU-Net [9]	40.6	59.0	55.2	49.0	38.0	17.1
FPN	39.4	60.1	55.6	48.3	35.6	12.9
Ours	41.6	59.9	55.8	49.5	39.3	19.6
Ours${}^{+}$	42.0	60.7	56.6	50.0	39.5	19.6

**Table 8:** Comparisons with other detectors on MS COCO test-dev. “MS${}_{train}$” denotes multi-scale training, otherwise using single-scale training. All experiments of our method set score_thr to 0.001, which slightly improves detection performance without a speed reduction. IoU-Aware R-CNN${}^{*}$ means that trainval dataset is used to train the detector and soft-NMS is employed at inference.

Method	Backbone	${\mathrm{MS}}_{\mathrm{train}}$	AP	${\mathrm{AP}}_{50}$	${\mathrm{AP}}_{75}$	${\mathrm{AP}}_{S}$	${\mathrm{AP}}_{M}$	${\mathrm{AP}}_{L}$
one-stage detectors								
SSD [12]	ResNet-101		31.2	50.4	33.3	10.2	34.5	49.8
RefineDet [30]	ResNet-101		36.4	57.5	39.5	16.6	39.9	51.4
RetinaNet [5]	ResNet-101		39.1	59.1	42.3	21.8	42.7	50.2
FSAF [17]	ResNet-101	✓	40.9	61.5	44.0	24.0	44.2	51.3
FSAF [17]	ResNeXt-101-64x4d	✓	42.9	63.8	46.3	26.6	46.2	52.7
FCOS [6]	ResNet-101	✓	41.5	60.7	45.0	24.4	44.8	51.6
FCOS [6]	ResNeXt-101-64x4d	✓	44.7	64.1	48.4	27.6	47.5	55.6
FoveaBox [31]	ResNet-101	✓	40.8	61.4	44.0	24.1	45.3	53.2
FoveaBox [31]	ResNeXt-101	✓	42.3	62.9	45.4	25.3	46.8	55.0
LTM [32]	ResNeXt-101-64x4d	✓	44.9	64.7	48.3	26.9	47.8	55.8
ATSS [33]	ResNeXt-101-32x8d	✓	45.1	63.9	49.1	27.9	48.2	54.6
two-stage detectors								
Faster R-CNN [7]	ResNet-101		34.9	55.7	37.4	15.6	38.7	50.9
Faster R-CNN w/FPN [11]	ResNet-101		36.2	59.1	39.0	18.2	39.0	48.2
Mask R-CNN [34]	ResNeXt-101		39.8	62.3	43.4	22.1	43.2	51.2
Libra R-CNN [20]	ResNet-101		41.1	62.1	44.7	23.4	43.7	52.5
Libra R-CNN [20]	ResNeXt-101-64x4d		43.0	64.0	47.0	25.3	45.6	54.6
Grid R-CNN [35]	ResNet-101		41.5	60.9	44.5	23.3	44.9	53.1
Faster R-CNN w/ PISA [21]	ResNeXt-101		42.3	62.9	46.8	24.8	45.5	53.1
Cascade R-CNN [8]	ResNet-101		42.8	62.1	46.3	23.7	45.5	55.2
TridentNet [36]	ResNet-101	✓	42.7	63.6	46.5	23.9	46.6	56.6
IoU-Aware R-CNN	ResNet-50		40.7	59.8	44.0	22.9	43.5	51.2
IoU-Aware R-CNN	ResNet-101		42.3	61.3	45.7	23.3	45.5	54.5
IoU-Aware R-CNN	ResNeXt-101-32x4d		43.4	62.8	46.8	24.7	46.7	55.1
IoU-Aware R-CNN${}^{*}$	ResNeXt-101-32x4d		44.3	62.9	48.3	25.6	47.5	56.5

## Data Availability

MS COCO: https://cocodataset.org/ (accessed on 5 June 2021); PASCAL VOC: https://host.robots.ox.ac.uk/pascal/VOC/ (accessed on 5 June 2021).
